# HOTAIR Up-Regulation Activates NF-κB to Induce Immunoescape in Gliomas

**DOI:** 10.3389/fimmu.2021.785463

**Published:** 2021-11-23

**Authors:** Yunfei Wang, Kaikai Yi, Xing Liu, Yanli Tan, Weili Jin, Yansheng Li, Junhu Zhou, Hongjun Wang, Chunsheng Kang

**Affiliations:** ^1^ Laboratory of Neuro-Oncology, Department of Neurosurgery, Tianjin Neurological Institute, Tianjin Medical University General Hospital, Tianjin, China; ^2^ Key Laboratory of Post-Neuro Injury Neuro-Repair and Regeneration in Central Nervous System, Ministry of Education and Tianjin City, Tianjin, China; ^3^ Department of Neuro-Oncology and Neurosurgery, Tianjin Medical University Cancer Institute and Hospital, Tianjin, China; ^4^ National Clinical Research Center for Cancer, Key Laboratory of Cancer Prevention and Therapy of Tianjin, Tianjin Clinical Research Center for Cancer, Tianjin, China; ^5^ Beijing Neurosurgical Institute, Capital Medical University, Beijing, China; ^6^ Department of Pathology, Hebei University Medical College, Baoding, China; ^7^ Department of Pathology, Affiliated Hospital of Hebei University, Baoding, China; ^8^ Department of Neurosurgery, The Second Affiliated Hospital of Harbin Medical University, Harbin, China

**Keywords:** HOTAIR, chromatin structure, NF-κB activation, immunoescape, inflammatory signaling pathway

## Abstract

**Background:**

Checkpoint blockade therapies targeting programmed death ligand 1 (PD-L1) and its receptor programmed cell death 1 promote T cell-mediated immune surveillance against tumors and have been associated with significant clinical benefit in cancer patients. The long-stranded non-coding RNA HOTAIR is highly expressed and associated with metastasis in a variety of cancer types and promotes tumor metastasis at least in part through association with the PRC2 complex that induces redirection to hundreds of genes involved in tumor metastasis. Here, we report that HOTAIR is an activator lncRNA of the NF-κB pathway and demonstrate that its apparent upregulation promotes inflammatory signaling and immune escape in glioma cells.

**Methods:**

Bioinformatics analysis was used to elucidate the relationship between HOTAIR and NF-κB pathway in HOTAIR knockdown glioma cells. At the cytological level, protein hybridization and immunofluorescence were used to detect the response of proteins in the NF-κB signaling pathway to HOTAIR regulation. ChIP and ChIRP experiments identified HOTAIR target genes. Animal experiments verified alterations in inflammation and immune escape following HOTAIR knockdown and activity inhibition.

**Results:**

HOTAIR activated the expression of proteins involved in NF-κB, TNFα, MAPK and other inflammatory signaling pathways. In addition, HOTAIR induced various proteins containing protein kinase structural domains and promoted the enrichment of proteins and complexes of important inflammatory signaling pathways, such as the TNFα/NF-κB signaling protein complex, the IκB kinase complex, and the IKKA-IKKB complex. In addition, HOTAIR aberrantly activated biological processes involved in glioma immune responses, T-cell co-stimulation and transcription initiation by RNA polymerase II. HOTAIR facilitated the induction of IκBα phosphorylation by suppressing the expression of the NF-κB upstream protein UBXN1, promoting NF-κB phosphorylation and nuclear translocation. *In vivo*, reduction of HOTAIR decreased PD-L1 protein expression, indicating that cells are more likely to be targeted by immune T cells.

**Conclusion:**

In conclusion, our results provide convincing evidence that lncRNA HOTAIR drives aberrant gene transcription and immune escape from tumor cells through the NF-κB pathway.

## Introduction

The role of the immune system in carcinogenesis remains unclear. Checkpoints are negatively regulated pathways in the immune regulatory system and the malignant evolution of tumors effectively suppresses the immune response by activating the expression of checkpoints that enable them to actively evade detection. Of the many checkpoint proteins, cytotoxic T lymphocyte protein 4 (CTLA4) and programmed cell death protein 1 (PD-1) have been the most studied. PD-1 is a cytokine receptor on the surface of T cells that is expressed on the surface of activated T cells and is capable of binding to two ligands, PD-L1 or PD-L2. Many cell types express PD-L1, including tumor cells and immune cells regulated by cytokines such as interferon (IFN)-γ. The binding of PD-1 to PD-L1 or PD-L2 impairs T-cell activity (1). In a study of brain tumor samples from 284 clinical glioma cases, Han et al. found that the ratio of CD4(+) tumor-infiltrating lymphocytes to CD8(+) tumor-infiltrating lymphocytes was inversely correlated with overall survival and that high expression of the transcription factor FoxP3 by regulatory T cells could be found in high-grade gliomas, whereas it was not observed in low-grade gliomas (2). In addition, tumor shrinkage was evident in high-grade gliomas after treatment with nivolumab, suggesting that, similar to other solid tumors, immunotherapy could be applied to the treatment of gliomas (3).

HOX antisense intergenic RNA (HOTAIR), originally identified by Rinn et al. in 2007, is an lncRNA that is overexpressed in a variety of human cancers (4). It located in the HOXC cluster on chromosome 12, and regulates the HOXD gene cluster on chromosome 2 (5). HOTAIR silences the transcription of genes located in distant chromosomal regions at the epigenetic level by interacting with the polycomb protein complex 2 (PRC2). This interaction allows the Zeste 2 enhancer (EZH2) to occupy specific gene themes, such as the HOXD theme and undergo H3K27me3 (trimethylation of histone H3 lysine K27) modification, leading to gene repression (6, 7). Other interchromosomal targets of HOTAIR include cancer-related genes such as pro-calmodulin (PCDH), hepatic ligand protein receptor (EPHA1) and NF-κB inhibitory protein IκBα (5, 8). Although epigenetic processes have been reported to have a key role in immunosuppression, including aberrant histone methylation induced by EZH2 (9–12), the direct role of HOTAIR in immunosuppression has not been investigated. However, enhanced HOTAIR activation has been observed in solid tumors, including mesenchymal glioma (13–15). Furthermore, according to the TCGA transcriptional classification scheme, PD-L1 appears to be expressed more in the mesenchymal subtype and may contribute as a potential marker for the mesenchymal subtype of glioblastoma (16), suggesting a potential interaction between PD-L1-dependent immunosuppression and HOTAIR.

As a widely expressed transcription factor with a rapid response, NF-κB regulates the cellular immune response and inflammatory signaling pathway under cytokine stimulation, and its downstream regulatory genes include a variety of immune-related cytokines. Following these stimuli, NF-κB inhibitor α (IκBα), which is coupled to NF-κB, is phosphorylated and then degraded by proteases. NF-κB is thus released and translocated to the nucleus, where it activates the expression of target genes. The underlying inflammation due to the presence of a tumor leads to aberrant NF-κB activation and promotes cancer invasion and metastasis, and such a phenomenon has been observed in many tumor types, including lung cancer, breast cancer, and glioma (17–19). In previous studies (20), ubiquitin regulatory X (UBX) domain-containing protein UBXN1 interacts with inhibitor of apoptosis proteins (cIAPs), the E3 ubiquitin ligase of RIP1 in the TNFα receptor complex, blocks cIAP1 recruitment to TNFα receptor 1 (TNFR1) and sequentially inhibits RIP1 polyubiquitin chain modifications. This is important for the recruitment of TGFβ-activated kinase 1 (TAK1) and TAK1-binding protein 2/3 (TAB2/3) heterodimeric complexes and IKK heterodimeric complexes. Activated TAK1 phosphorylates and activates IKKβ, which phosphorylates IκBα and leads to its degradation and NF-κB nuclear translocation (21, 22)

In our ongoing efforts to understand the biological and pathological role of lncRNA, a previous sequencing analysis of HOTAIR-related proteome and clinical samples determined that HOTAIR activates a variety of proteins containing protein kinase domains and promotes the enrichment of important inflammatory signaling pathway proteins and their complexes, such as the TNFα/NF-κB signaling protein complex, I-kappaB kinase complex, and IKKα-IKKβ complex. Moreover, HOTAIR upregulates the expression of several inflammatory signaling proteins, such as TNFα, and MAPK. An analysis of clinical samples found abnormal HOTAIR expression in stromal glioblastoma, which was correlated with the malignancy degree of the glioma. HOTAIR abnormally activated biological processes involved in the immune response, T cell synergistic stimulation, and RNA polymerase II transcription initiation in glioma. Simultaneously, HOTAIR was highly positively correlated with the tumor immune escape related protein PD-L1; furthermore, it was negatively correlated with the expression of NFκB repressing factor (NKRF), Protein Kinase C Theta (PRKCQ) and ubiquitin-associated and ubiquitin regulatory X domain-containing protein 1 (UBXN1), which are repressors of the NF-κB signaling pathway. Therefore, we concluded that HOTAIR might regulate the gene transcription of key repressors of the NF-κB activation pathway through epigenetic modulation, thereby participating in the immune escape biological process in glioma cells.

## Materials and Methods

### Cell Lines and Culture Conditions

The human epithelial glioma cell line U87 MG was obtained from the American Type Culture Collection (ATCC, Manassas, VA, USA) in May 2015. Its short tandem repeat (STR) profile is presented on the ATCC website (https://www.atcc.org/products/all/HTB-14.aspx#specifications); furthermore, we performed a reauthentication by STR analysis in June 2017. The primary cell line TBD was derived from a GBM patient with recurrent lesions undergoing surgery at the Hebei University Affiliated Hospital according to a protocol reported by Dong et al. (23). After the three cell lines were obtained, they were immediately proliferated, amplified, and frozen in liquid nitrogen. All cells used in subsequent experiments were recovered from these cryopreserved cell lines. The U87 MG and TBD cells were cultured in Dulbecco’s modified Eagle’s medium (DMEM) containing 10% fetal bovine serum (FBS). The U87 cells were cultured in DMEM/F12 supplemented with 10% FBS. All cells were proliferated at 37°C in a humidified atmosphere of 5% CO_2_. All glioma cells with the exception of *in vivo* cultures were maintained for less than eight passages. Cells in the logarithmic growth phase or at 80% confluence were used for subsequent experiments.

### Cell Culture and Labeling

To profile protein expression in response to HOTAIR knockdown, U87 cells transfected with lentivirus-siRNA of HOTAIR (siHOTAIR-U87) or a negative control (siNC-U87) were labeled with the SILAC Protein Quantitation Kit (Invitrogen, Carlsbad, CA) according to manufacturer’s instructions. siHOTAIR-U87 cells and siNC-U87 cells were cultured separately in DMEM supplemented with 10% FBS and either the light isotopic forms of [U-^12^C_6_]-L-lysine and $\left[\text{U}{-}^{12}{\text{C}}_{6}^{14}{\text{N}}_{4}\right]$ -L-arginine or the heavy isotopic forms of [U-^13^C_6_]-L-lysine and $\left[\text{U}{-}^{13}{\text{C}}_{6}^{15}{\text{N}}_{4}\right]$ -L-arginine. After six passages, the heavy labeling efficiency of [U-^13^C_6_]-L-lysine and $\left[\text{U}{-}^{13}{\text{C}}_{6}^{15}{\text{N}}_{4}\right]$ -L-arginine in control cells was evaluated by mass spectrometry to confirm a greater than 99% labeling efficiency. Then, cells were continuously expanded in SILAC medium until reaching the desired confluence. Finally, cells from both the heavy- or light-labeled pool were harvested separately and combined in equal amounts.

Nondenatured protein lysates were obtained from the harvested cells and were centrifuged to collect protein precipitates. The collected fractions were combined, vacuum-dried, and analyzed by high-performance liquid chromatography with tandem mass spectrometry (HPLC-MS/MS).

### Proteomic and Transcriptome Profiling

Based on the SILAC labeling-based proteomics, we comparatively quantified the host proteome of siHOTAIR-U87 and siNC-U87 cells. We analyzed the quantifiable proteome dataset for three enrichment gene ontology (GO) categories: Biological Process, Protein Complex, and Protein Domains. Pathway clustering was conducted using the KEGG. Fisher’s exact/chi-squared tests and false discovery rates (FDRs) were used to determine significance, denoted by the *P*-value between the GO term and the pathway correlated to the experimental conditions. Lower FDRs indicated smaller errors in the determination of the *P*-value.

Triplicate RNA samples from four sets of U87 cells transfected with lenti-HOTAIR or lenti-NC for 48 h were randomly selected for RNA-sequencing. For mRNA network analysis, biological process (BP) analysis through gene ontology and pathway clustering through KEGG were performed. Furthermore, two large cohorts were used for gene ontology and correlation analysis: the Chinese Glioma Genome Atlas cohort (CGGA, http://www.cgcg.org.cn/, n=301) and The Cancer Genome Atlas cohort (TCGA, https://tcga-data.nci.nih.gov/docs/publications/gbm_2013/, including the transcriptome of 164 RNA samples profiled by RNA-seq).

### RNA Extraction and RT-PCR

Total RNA was isolated from cells using the TRIzol reagent (Invitrogen). Next, 1 μg total RNA was reverse transcribed into cDNA using the GoScript™ reverse transcription system (Promega, USA) according to the manufacturer’s instructions. Analyses were performed utilizing PowerUp™ SYBR™ Green on a ABI QuantStudio 5 real-time fluorescence detection PCR system. The expression levels of specific genes are reported as ratios to GAPDH expression in the same master reaction. The primers used for real-time RT-PCR are listed in the **Supplementary Table 1**.

### Luciferase Reporter Assays

Dual-luciferase assays were performed 48 h post-transfection following the manufacturer’s protocol (Dual-Luciferase Reporter Assay System, Promega, USA), and expression was detected with a microplate reader (Synergy2, BioTek, USA). All cell lines were transfected with 0.1 µg of the luciferase reporter p-NF-κB-Luc plus 0.01 µg of the Renilla reporter pRL-TK. After AQB treatment for 48 h, the transfected cells were collected. The luciferase assays were performed using a dual-specific luciferase assay kit (Promega, USA).

### T Lymphocyte Purification and Flow Cytometry

Female C57BL/6 mice (8-10 weeks old) with GL261-induced tumor xenografts were treated parenterally with DMSO or AQB and were used for T lymphocyte purification and flow cytometry. The lymph nodes were removed aseptically, minced, and suspended in RPMI 1640 medium. Single cell suspensions were prepared *via* filtration through a sterile sieve mesh. Blood was collected from the eyeballs of C57BL/6 mice, and heparin was added for anticoagulation. Erythrocytes were lysed with a red blood cell lysis buffer, and the cells were washed twice with cold PBS supplemented with 2% FBS. After washing, T lymphocytes harvested from spleens and blood were stained with PE-conjugated-CD8α, FITC conjugated-CD4, and APC conjugated-CD3 (eBioscience). The samples were acquired using an LSR II (BD Biosciences) flow cytometer, and the data were analyzed with Kaluza Analysis Software (Beckman Coulter, Brea).

### Laser Confocal Immunofluorescence Assay

Cells seeded on glass-covered slides were fixed with 4% paraformaldehyde, permeabilized with 0.25% Triton X-100, and blocked with 1% bovine serum albumin (BSA). Next, the cells were probed with primary antibodies diluted in 1% BSA solution overnight at 4°C. Rabbit or mouse IgG antibodies coupled with Alexa (1:100 dilution, Invitrogen) were used as secondary antibodies followed by nuclear staining with DAPI. The images were visualized and captured by laser confocal scanning microscopy (Olympus, Tokyo, Japan). Antibodies against human NF-κB, pNF-κB, H3K27me3 (1:100 dilution, Cell Signaling Technology), and PD-L1 (1:1000, Abcam) were utilized as primary antibodies.

### Western Blot Analysis

Western blot was performed as described previously. Thirty µg protein were loaded per lane. Cell lysates were separated on analytical 10% SDS-PAGE gels and transferred onto poly vinylidene difluoride (PVDF) membranes. Non-specific binding was blocked by incubation with 5% skim milk. Antibodies against human NF-κB, pNF-κB, H3K27me3, IKKα, IKKβ, IκBα, pIKKα, pIKKβ (1:1000 dilution, Cell Signaling Technology), PD-L1 (1:1000, Abcam), and GAPDH (1: 4000 dilution, Proteintech) were used as primary antibodies.

Rabbit or mouse IgG antibodies coupled to horseradish peroxidase (1:10000 dilution, Promega) were utilized as secondary antibodies. Antibody-labeled protein bands on the membranes were detected with a G:BOXiChemi XT chemiluminescence and fluorescence imaging system (Syngene). Band densities were determined using the Image J software.

### Chromatin Immunoprecipitation (ChIP)

ChIP assays were performed using the EZ-ChIP kit (Millipore). Briefly, cells were fixed with 1% formaldehyde, and then formaldehyde was neutralized with 0.125 M glycine. Cells were then lysed with SDS and protease inhibitor lysis buffer. Soluble chromatin of 200-1000 bp was collected after sonication, pre-cleared in 1:10 dilution buffer, and incubated overnight with IgG, H3K27me3, and NF-κB antibodies in a rotating platform at 4°C. After washing the immune complexes captured by G-Sepharose protein beads, the bound DNA fragments were eluted and analyzed by real-time quantitative pcr using ChIP primers. The primers used for real-time RT-PCR are listed in the **Supplementary Table 2**.

### Chromatin Isolation by RNA Purification (ChIRP)

The ChIRP assay was performed using the Magna ChIRP RNA Interactome Kit (Millipore, USA) following the manufacturer’s guidelines. Briefly, a total of 1 × 10^7^ cells was lysed in complete lysis buffer for each reaction, and the DNA was then sheared into small fragments through sonication. Then the lysate was incubated with biotin-labeled probes that could hybridize with HOTAIR or control probe. Finally, the probes were extracted by streptavidin magnetic beads, the combined protein was isolated for immunoblotting analysis, and the RNAs and DNAs samples were enriched for agarose electrophoresis. The probes used are listed in the **Supplementary Table 3**.

### Tumor Xenografts

Bagg albino (BALB)/c nude mice (4 weeks old) and C57BL/6 black mice (4-6 weeks old) were purchased from the Animal Center at the Cancer Institute of the Chinese Academy of Medical Science (Beijing, China). All experimental protocols were approved by the Tianjin Medical University Animal Care and Use Committee. To establish orthotopic models, glioma cells transduced with a luciferase lentivirus were implanted stereotactically: anthropogenic U87 or TBD in BALB/c nude mice and murine GL261 in C57BL/6 black mice. The mice were randomly assigned into six groups (n = 8 per group). Twenty-one days after implantation, PBS or AQB (5 mg/kg) was administered by intraperitoneal injection every other day for 14 days. The nude mice were imaged for luciferase activity once per week. The mice were sacrificed on day 42, and their brains were removed for paraffin-embedding. The ratio of CD4/CD8 T cells was measured in blood and lymph node specimens of black mice, and the brain tissues were used to prepare paraffin sections for immunohistochemical and hematoxylin-eosin staining.

### Immunohistochemistry

Brain tumor tissues in paraffin were sectioned at 4 μm and mounted on slides. Paraffin slices were dewaxed and incubated with primary antibodies. Primary antibodies against human NF-κB, pNF-κB (1: 200 dilution, Cell Signaling Technology), and PD-L1 (1:200, Abcam) were used for antigen recognition; secondary antibodies were conjugated with biotin labels. DAB was utilized as a developing method to detect the biotin reporter, and hematoxylin was utilized for counterstaining. Next, sections were rinsed with PBS, re-stained with ammonia, stained with hematoxylin, and observed by microscopy. The results were analyzed using the National Institutes of Health (NIH) ImageJ software.

### Hematoxylin-Eosin Staining

Paraffin-embedded tissue sections were subjected to hematoxylin-eosin staining. After blocking with 3% H_2_O_2_ and non-immune rabbit serum, sections were incubated with primary antibodies (1:200 dilutions) overnight at 4°C followed by an incubation with a streptavidin-biotinylated secondary antibody (ZSGB-BIO) for 1 h at 37°C. The chromogenic substrate used was 3,3’-diaminobenzidine, and hematoxylin was utilized as the counterstain. The sections were visualized under a light microscope.

### Statistical Analyses

Statistical analyses were performed utilizing Kaplan-Meier survival curves, the long-rank test, and univariate and multivariate Cox proportional hazards regression analysis with SPSS 16.0 (IBM, USA). All values are presented as the mean ± standard deviation (SD). Statistical comparisons between two groups were performed using Student’s *t*-test. Differences among three or more groups were determined by two-way ANOVA followed by Dunnett’s test. Significance was set to *P < 0.05, **P < 0.01.

## Results

### Proteomics Analysis Revealed That HOTAIR Regulates Proteins in Glioma Cells

**Figure 1:**
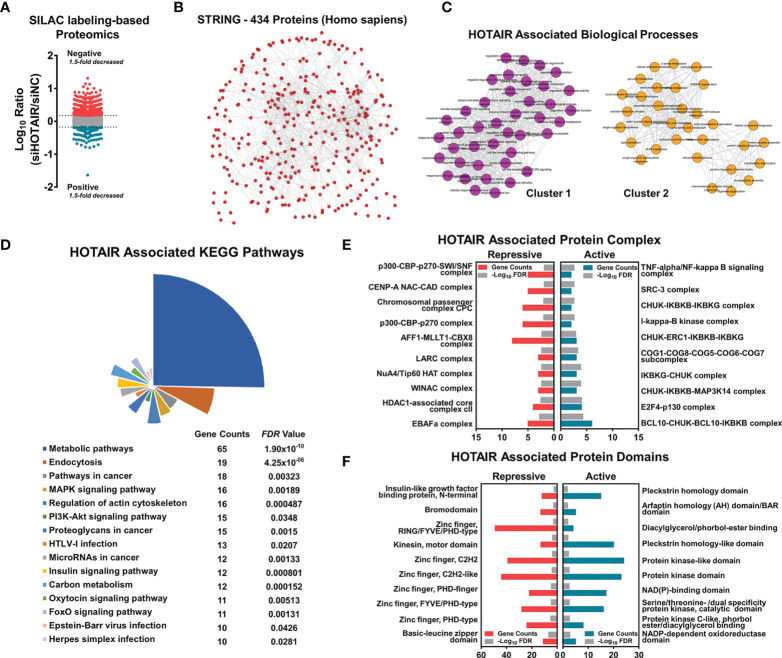
HOTAIR regulated protein level associated with multiple biological progress, when applied to SILAC labeling-based proteome of U87 cells. **(A)** The scatter plot shows the number of differential proteins detected by proteome sequencing, with a difference ratio greater than 1.5. **(B)** STRING protein network shows that 434 proteins had high correlation. **(C–F)** Enrichment and clustering analysis of the quantifiable proteomics data set including HOTAIR associated biological processes clustering **(C)**, KEGG pathway analysis **(D)**, protein complex enrichment **(E)** and protein domains enrichment **(F)**.

An abnormally high expression of the lncRNA HOTAIR has been previously demonstrated in glioma cells. HOTAIR regulates genes by anchoring epigenetic modification proteins and causes abnormalities in multiple signaling pathways (5, 13, 24, 25). We knocked down HOTAIR in glioma cells by siRNA with SILAC labeling, and then total protein was extracted for proteome mass spectrometry. A proteomics analysis showed that in HOTAIR knockdown glioma cells, 1074 proteins were up-regulated, whereas 390 proteins were inhibited (**Figure 1A**, **Supplementary Table 4**). Among these proteins, 434 protein correlations were enriched in one protein network (**Figure 1B**). HOTAIR was associated with two main cluster networks of biological processes (**Figure 1C**, **Supplementary Table 5**). Next, proteins were annotated with the Kyoto Encyclopedia of Genes and Genomes (KEGG), and a significant association of HOTAIR with proteins in metabolic pathways and in the MAPK and PI3K-Akt signaling pathways was identified (**Figure 1D**). An InterPro database protein complex analysis demonstrated that proteins annotated in complexes with histone acetyltransferases were repressed by HOTAIR, including the p300-CBP-p270-SWI/SNF, CENP-A NAD-CAD, and NuA4/Tip60 HAT complexes with enrichment *P* values 0.001765, 0.003027, and 0.011090, respectively. HOTAIR activated the TNF-α/NF-κB, CHUK-IKBKB-IKBKG, and IκB kinase complexes, which are involved in the NF-κB signaling activation process, with enrichment *P* values 0.000053, 0.000131, and 0.000131, respectively (**Figure 1E**). To investigate the HOTAIR-associated proteins domains, we analyzed their profiles in the CORUM database. The expression of proteins containing an IGF binding domain, a bromodomain, and a zinc finger was repressed by HOTAIR (*P* values 0.003775, 0.001489, and 0.001130, respectively). Proteins activated by HOTAIR mostly contained a protein kinase domain or a kinase-like domain (enrichment *P* values 0.003446 and 0.006261, respectively) (**Figure 1F**).

**Figure 2:**
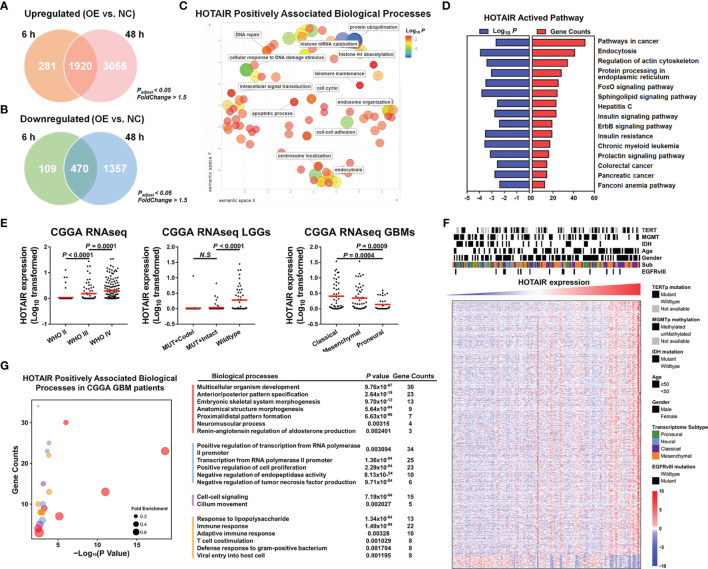
HOTAIR regulated gene expression participated in multiple biological processes in different dataset. **(A)** HOTAIR upregulated 2201 genes after 6h expressing in U87 and 4975 genes after 48h, respectively, and there were 1920 overlapping genes. **(B)** HOTAIR downregulated 579 genes and 1827 genes when HOTAIR were expressed for 6h and 48h in U87 respectively, and the overlapping genes reached 470. **(C, D)** HOTAIR regulated genes were enriched in several biological processes **(C)** and pathways **(D)**. **(E)** HOTAIR expression in CGGA RNAseq dataset (n=301). **(F)** Cluster heatmap showed the gene expression level correlated with HOTAIR expression in glioma based on CGGA RNAseq dataset (n=301). **(G)** Quantifiable genes of GBM patients in CGGA RNAseq dataset associated with HOTAIR were classified by ontology annotation based on biological processes.

**Figure 3:**
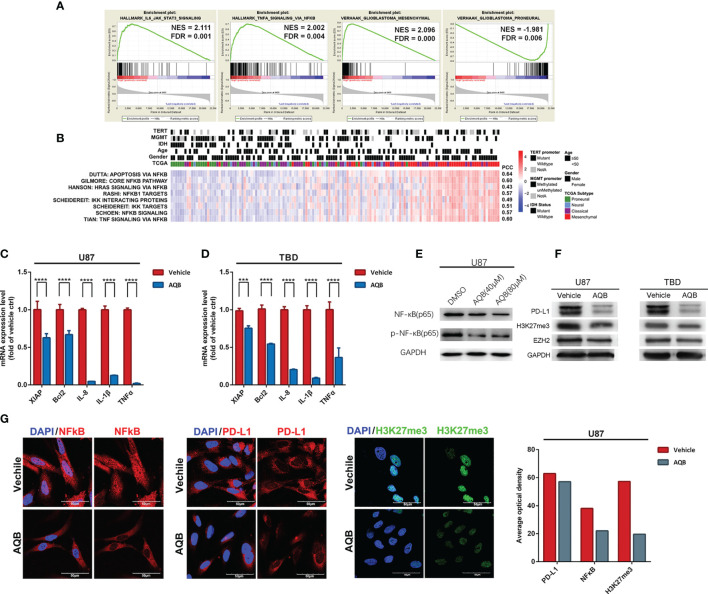
HOTAIR may regulate gene expression pattern in an NF-κB-dependent manner in glioma. **(A)** The enrichment plots of gene expression signatures of HOTAIR-induced IL6-JAK-STAT3 signaling and TNFα signaling *via* NF-κB, and the signatures of samples annotated in mesenchymal glioma and proneural glioma, sorted according to the differences between the samples with high and low level HOTAIR expression. **(B)** Enrichment heatmap showed the NFκB-dependent biological processes associated with the grade of samples. **(C, D)** NF-κB regulated gene expression levels in U87 **(C)** and TBD **(D)** at 24 hours after treatment with AQB. Values are means ± s.d. from n = 3 independent experiments. The *P* value was determined by two-sided Student’s t-test. **(E)** Immunobolts detect the protein level of NF-κB and pNF-κB, using GAPDH as control in U87 cells treated with AQB at the concertration of 40μM and 80μM. **(F)** Immunoblots showing the effect of AQB on expression of PD-L1 and EZH2 in U87 and TBD cells, using GAPDH as control. **(G)** Immunoflourescence showing the protein level and protein distribution of H3K27me3, NF-κB and PD-L1 in DMSO or AQB treated U87 cells. The bar chart indicates differences in the mean optical densities of NF-κB, PD-L1, and H3K27me3 proteins in DMSO or AQB-treated U87 cells. Scale bar, 50μm. ***P < 0.001, ****P < 0.0001.

To elucidate HOTAIR’s effects, we analyzed different databases and datasets including samples from glioma cell lines and clinical glioma samples. Microarray mRNA datasets, in which HOTAIR was overexpressed for 6 h or 48 h in U87 glioma cells, exhibited 1920 upregulated and 470 downregulated genes at both timepoints (**Figures 2A, B**). HOTAIR was positively associated with biological processes, including cell cycle and intracellular signal transduction and with multiple pathways (**Figures 2C, D**). A CGGA database analysis based on 301 patients revealed high HOTAIR expression in WHO III and IV grade glioblastoma samples, especially in classical and mesenchymal glioblastomas (**Figures 2E, F**). Quantifiable genes associated with HOTAIR in glioblastoma samples in the CGGA RNAseq dataset were classified by an ontology annotation based on biological processes. The findings demonstrated that HOTAIR regulates the immune response, T cell co-stimulation, and adaptive immune response (**Figure 2G**). As shown in **Figure 3A**, multiple genes which were participated in IL6-JAK-STAT3 signaling and TNFα/NF-κB signaling pathways, significantly enriched in HOTAIR positively correlated gene group. The expression of HOTAIR was positively correlated with the messenchymal subtype of glioma, but negatively correlated with the subtype of proneural (**Figure 3A**). Similar conclusions can also be drawn from the sequencing data published by several teams that the activity of NF-κB and downstream pathways is significantly positively correlated with the malignant messenchymal subtype of glioma (**Figure 3B**).

### AQB Inhibited Inflammatory Signaling Pathways and the Checkpoint Protein Downstream of NF-κB

To verify the database analysis results regarding a relationship between HOTAIR and NF-κB, the regulatory effect of HOTAIR on the NF-κB pathway was verified by *in vitro* experiments. In previous study, we identified a small molecule compound AC1Q3QWB (AQB) as an efficient disruptor of HOTAIR-EZH2 interaction, resulting in blocking of PRC2 recruitment (14, 26). First, we treated U87 and TBD human glioma cells with AQB. We found that AQB significantly reduced the levels of the inflammatory associated proteins including XIAP, Bcl2, IL-8, IL-1β and TNFα, compared with DMSO treatment in the control group (**Figures 3C, D**). After treatment with 40uM and 80uM AQB, there was a subtle decrease in the total amount of NF-kB, especially after 80μM AQB treatment, while the level of phosphorylated NF-kB decreased significantly (**Figure 3E**). Furthermore, PD-L1 expression was inhibited by AQB. With AQB interfering with the EZH2/HOTAIR combination, H3K27me3 levels decreased, but the EZH2 protein level did not change significantly (**Figure 3F**). The same phenomenon can be significantly observed in the immunofluorescence staining experiment, PD-L1, nuclear NF-κB and H3K27me3 in U87 cells treated with AQB decreased significantly (**Figure 3G**). This suggests that HOTAIR functional inhibition can regulate the downstream of the NF-κB signaling pathway.

### The Stock of HOTAIR Is Positively Associated to the Positioning and Function of NF-κB

**Figure 4:**
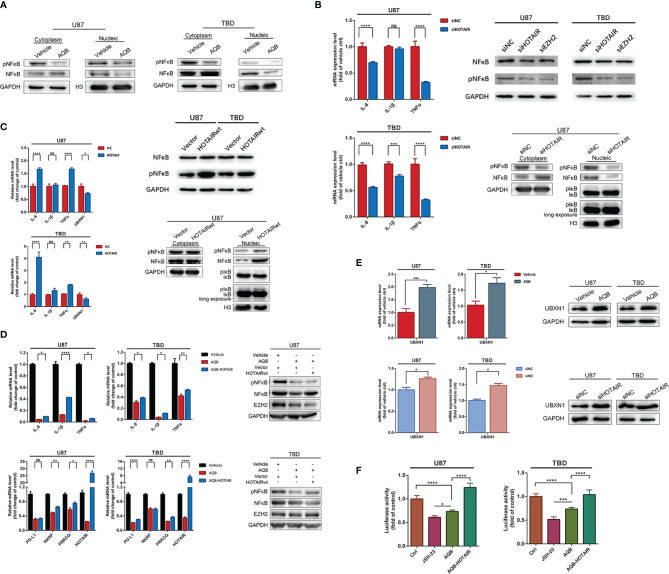
Inhibition of H3K27me3 modification can regulate the activation and function of NF-κB. **(A)** Immunoblots showing the distribution of NF-κB and pNF-κB proteins in the nucleus and cytoplasm, with GAPDH as the cytoplasmic protein control and histone H3 as the nuclear protein control. **(B)** Quantitative PCR to detect mRNA expression levels of IL-8, IL-1β, and TNFα in U87 and TBD cells treated with HOTAIR knockdown. The protein expression levels of NF-κB and pNF-κB in U87 and TBD cells with HOTAIR knockdown and EZH2 knockdown were detected by immunoblots. **(C)** Quantitative PCR to detect mRNA expression levels of IL-8, IL-1β, TNFα and UBXN1 in U87 and TBD cells overexpressing HOTAIR. Immunoblots detected the protein expression levels of NF-κB and pNF-κB in U87 and TBD cells overexpressing HOTAIR. **(D)** Quantitative PCR detecting mRNA expression levels of IL-8, IL-1β, TNFα, PD-L1, NKRF, PRKCQ and HOTAIR in U87 and TBD cells treated with AQB or AQB deals with joint HOTAIR overexpression. Immunoblots detected the protein expression levels of NF-κB and pNF-κB in U87 and TBD cells treated with AQB or AQB deals with joint HOTAIR overexpression. **(E)** Quantitative PCR and immunoblots showing the message RNA and protein level of UBXN1 in U87 and TBD cells treated with AQB using vehicle as control. **(F)** NFκB function was analysis using NFκB binding domain fused luciferase reporter vector in U87 and TBD cells treated with JSH-23, or AQB or AQB joint HOTAIR overexpression. For all the statistical histograms above, values are means ± s.d. from n = 3 independent experiments. The *P* value was determined by two-sided Student’s t-test. ns, not statistically significant, *P < 0.05, **P < 0.01, ***P < 0.001, ****P < 0.0001.

To test the effect of HOTAIR on NF-κB, we used AQB to inhibit the function of HOTAIR or varied the stock of HOTAIR using distinct siRNAs or lenti-HOTAIR virus. In U87 and TBD cells treated with DMSO or 40μM AQB, fewer NF-κB proteins entered into the nucleus, while phosphorylated-NF-κB proteins in the cytoplasm and nucleus decreased (**Figure 4A**). Knocking down HOTAIR through distinct siRNAs caused decreased mRNA expression of IL-8 and TNFα in both U87 and TBD cells. It caused decreases in phosphorylated NF-κB, which is the same as knocking down EZH2 (**Figure 4B**). In siHOTAIR U87 cells, western blotting showed that NF-κB in the cytoplasm increased, but less in the nucleus. Correspondingly, phosphorylated NF-κB decreased in both the cytoplasm and the nucleus. And the phosphorylated IκB decreased in nuclear. (**Figure 4B**). Correspondingly, when we transferred HOTAIR overexpressed lentivirus into U87 and TBD cells, the level of phosphorylated NF-κB increased, the content of nuclear NF-κB protein and phosphorylated NF-κB protein increased, and the phosphorylated IκB increased. The mRNA of IL-8 and TNFα were all increased induced by overexpressed HOTAIR lncRNA (**Figure 4C**). It suggested that inhibiting the function of HOTAIR or reducing the stock of HOTAIR can reduce the phosphorylation modification of NF-κB, thereby affecting the expression of NF-κB regulatory protein. We then tested the results of overexpression of HOTAIR in glioma cells treated with AQB. mRNA expression level showed that HOTAIR could restore the expression of suppressed IL-8, IL-1β and TNFα. Western blotting showed that overexpression of HOTAIR in TBD cells could restore the level of phosphorylated NF-κB inhibited by AQB, but the effect was not significant in U87 (**Figure 4D**). This means that the combination of HOTAIR and EZH2 to regulate epigenetic modification is the key to regulating NF-κB phosphorylation.

Furthermore, in luciferase reporter assays detecting NF-κB transcription activity, AQB inhibited the transcriptional regulatory activity of NF-κB similarly to the NF-κB inhibitor JSH-23, and this inhibition was reversed by overexpression of HOTAIR (**Figure 4F**).

### HOTAIR Regulation on NF-κB Signal Pathway Through the Negative Regulator UBXN1

In order to further clarify the intermediate factors of HOTAIR regulating NF-κB pathway activation, we tested several factors involved in the regulation of NF-κB signaling pathway. NKRF encodes a transcriptional inhibitor that binds to negative regulatory elements to inhibit the transcriptional regulation of NF-κB (27). PRKCQ encodes a serine/threonine protein kinase that can activate the IKK complex, thus activating NF-κB resulting in nuclear translocation and activation (28, 29). As shown in **Figure 4D**, in U87 and TBD cells treated with AQB, the mRNA expression of NKRF and PRKCQ were both inhibited by AQB, suggesting that their gene expression may not be regulated by HOTAIR through H3K27 trimethylation. But we found another protein, UBXN1, which was reported as negative regulator of NF-κB pathway decreased in LV-HOTAIR treated U87 and TBD cells (**Figure 4C**). As shown in **Figure 4E**, both the mRNA and protein of UBXN1 were induced by AQB in U87 and TBD cells. These results suggest that UBXN1 could be a possible bridge for HOTAIR to regulate the activation of NF-κB.

**Figure 5:**
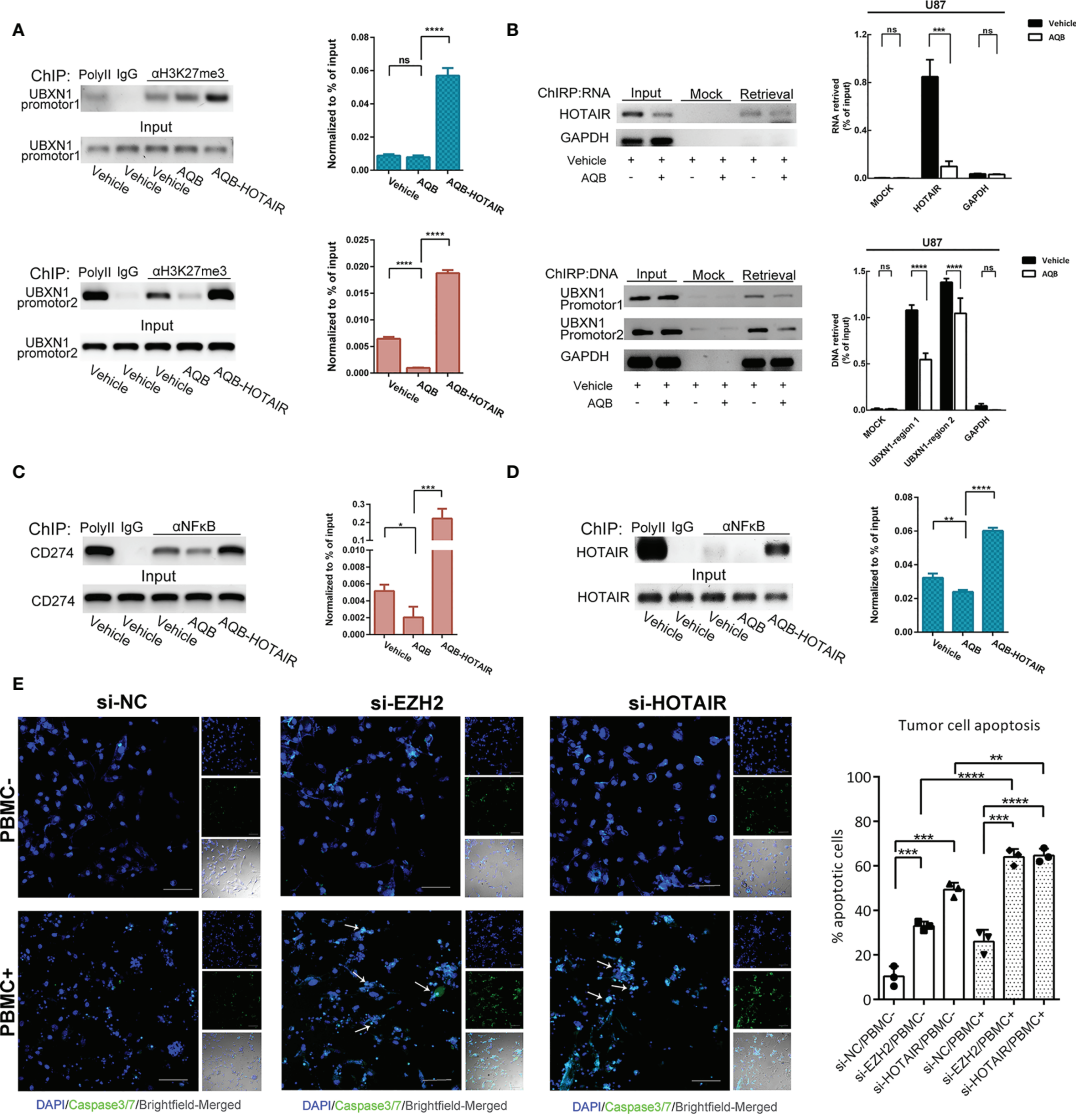
HOTAIR inhibited the expression of UBXN1 by epigenetic modification. **(A)** ChIP assay detected the H3K27me3 modification on the promotor region of *UBXN1.* **(B)** Upper: ChIRP assay enriches HOTAIR RNA with high yield and specificity. Lower: ChIRP assay detect the HOTAIR binding region on the promotor of *UBXN1*. **(C)** ChIP assay detected NF-κB binding on the promotor of *CD274* in U87 treated with AQB or vehicle as control. **(D)** ChIP assay detected NF-κB binding on the promotor of *HOTAIR* in U87 cells treated with AQB or vehicle as control. **(E)** T cell killing assay of TBD cells treated with siRNAs for EZH2 and HOTAIR. Cell nuclei were stained by DAPI in blue, and apoptotic cells were displayed by fluorescent products of caspase 3/7 cleavage (green). For all the statistical histograms above, values are means ± s.d. from n = 3 independent experiments. The *P* value was determined by two-sided Student’s t-test.

The modification of H3K27me3 in UBXN1 promoter region decreased when the function of HOTAIR was inhibited by AQB. On the contrary, overexpression of HOTAIR can restore the modification level of H3K27me3. It indicated that HOTAIR can directly regulate the expression level of UBXN1 by regulating histone modification (**Figure 5A**). In the ChIRP experiment, we observed that when AQB acted, HOTAIR bound to chromatin decreased, and HOTAIR bound to chromatin DNA in the UBXN1 promoter region decreased (**Figure 5B**).

### HOTAIR Regulation on PD-L1 Expression Affected T Cell-Dependent Toxicity

It has been demonstrated that PD-L1 on tumor cells is sufficient to mediate T cell tolerance, and inhibition of PD-L1 on tumor cells can effectively activate anti-tumor immunity (30). As the transcription regulation target of NF-κB, we found that the gene expression of PD-L1 was positively regulated by HOTAIR (**Figure 4D**). Moreover, a genome immune coprecipitation experiment revealed that AQB significantly inhibited NF-κB in the *CD274* gene promoter region, and that HOTAIR overexpression reversed and promoted NF-κB enrichment in this area, implying that AQB may regulate the immune sensitivity of tumor cells by inhibiting the transcriptional regulation of *CD274* gene by NF-κB (**Figure 5C**). Moreover, NF-κB recognized the promoter region of *HOTAIR*, suggesting that there might be regulatory feedback circuits involving lncRNA and transcription factors (**Figure 5D**).

To test whether the inhibition of PD-L1 mediated by HOTAIR affects the immune sensitivity of tumor cells, we co-cultured glioma cells treated with EZH2 siRNA or HOTAIR siRNA with activated human peripheral blood mononuclear cells (PBMCs). As expected, disruption of EZH2 or HOTAIR increased the sensitivity of tumor cells to T cell killing (**Figure 5E**). These results suggested that HOTAIR alters PD-L1 gene expression and T cell toxicity through NF-κB signaling pathway and highlight HOTAIR as a promising target for tumor immunotherapy.

### Blockade of HOTAIR Function Activated Anti-Cancer Immunity *In Vivo*

**Figure 6:**
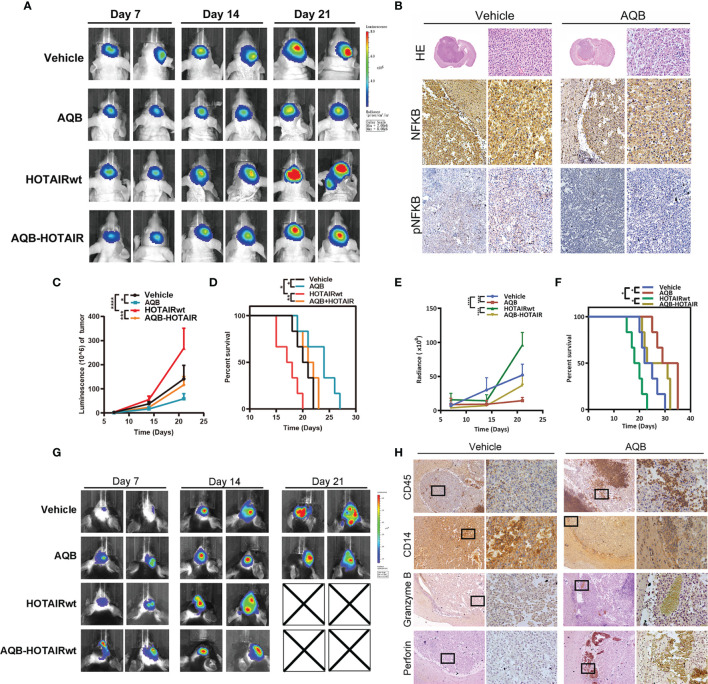
Apparent inhibition of UBXN1 expression levels activated tumor growth and tumor growth regulated by NF-κB *in vivo*. **(A)** Representative pseudocolor bioluminescence images of mice implanted with intracranial tumors infected with HOTAIR overexpressing lentivirus or control lentivirus, and heal every other day with vehicle or AQB. **(B)** Representative images of the H&E-stained mice brain tissue and the immunohistochemical staining of NF-κB and pNF-κB in brain tissues. Nuclei were stained by DAPI. **(C)** The tumor growth curve with time in xenotypic *in situ* tumor model animal was calculated by corresponding light value in **(A)**. **(D)** Kaplan-Meier survival plot shows the overall survival of glioblastoma TBD mice treated with regime describe previously. **(E)** In heterotypic *in situ* tumor-bearing black mice, the tumor growth curve over time was calculated from the corresponding light values in **(G)**. **(F)** Kaplan-Meier survival plot shows the overall survival of glioblastoma TBD mice with same treatment. **(G)** Representative pseudocolor bioluminescence images of tumor-bearing C57BL/6 mice as shown previously, and heal every other day with vehicle or AQB. **(H)** Representative images of the immunohistochemical staining of CD45, CD14, perforin and Granzyme B in brain tissues. For all the statistical collapsed line graph above, values are means ± s.d. from n = 3 independent experiments. The P value was determined by two-sided Student’s t-test.

To further confirm the regulation of HOATIR on PD-L1 dependent on the NF-kB pathway, we performed *in vivo* tests. We evaluated lentivirus-mediated HOTAIR overexpression and anti-HOTAIR AQB treatment alone or combination in TBD orthotopic tumor model established in Bagg albino (BALB)/c nude mice. AQB treatment resulted in a significant reduction of the intracranial tumor volume compared with the DMSO group(43% reduction with *P* < 0.05), contrarily, overexpression of HOTAIR increased the malignant progression of the tumor (52% enhancement in nude mice, *P <* 0.05; **Figures 6A, C**). Morphologically, hematoxylin-eosin-stained slides indicated that AQB induced shrinkage of tumor cell nuclei (**Figure 6B**). Compared with the control group (median survival: 19 days), AQB treatment group (median survival: 23 days) showed prolonged survival in Kaplan-Meier survival curves (*P* < 0.05; **Figure 6D**). The same results appeared in the HOTAIRwt group (median survival of HOTAIRwt: 17 days and HOTAIRwt-AQB: 21 days; **Figure 6D**). Moreover, immunohistochemistry staining showed the reduction in nuclear NF-κB and phospho-NF-κB levels in the AQB group (**Figure 6B**).

Next, we constructed TBD cell xenograft tumors in C57BL/6 black mice to established immunocompetent mouse model (**Figure 6G**). The *in vivo* bioluminescence imaging showed that AQB could dramatically inhibit the tumor growth of both the control group (72% reduction with *P* < 0.05) and the HOTAIRwt group (61% reduction with *P* < 0.05; **Figure 6E**). AQB treatment significantly prolonged the survival of glioma bearing mice both in control group and HOTAIRwt group (36.2% and 42% respectively; **Figure 6F**). Moreover, immunohistochemical staining showed that the infiltration of CD45 labeled leukocytes in tumor increased under AQB treatment, while the infiltration of CD14 labeled macrophages decreased, as well as the small amount of perforin and granzyme B staining was present in the tumor portion (**Figure 6H**). Taken together, these findings indicate that the inhibition of HOTAIR function blocks the expression of PD-L1 to a certain extent on the surface of glioma cells, thereby further promotes the infiltration of tumor-related immune cells. Collectively, these experiments supported the notion that HOTAIR silencing the expression of UBXN1 can regulate the activation of NF-kB pathway and induce the expression of checkpoint protein PD-L1 on surface of glioma cells, thereby initiating glioma to escape T cell recognition and killing.

## Discussion

HOTAIR expression is commonly altered in glioblastoma and is especially abnormally elevated in mesenchymal glioma. Furthermore, HOTAIR promotes tumor progression through activation of β-catenin, PKM2, and other pathways (13, 24, 25). We have previously demonstrated that HOTAIR is a positive factor in the progression of malignant glioma and is directly related to poor prognosis in glioma patients. Moreover, we have shown that HOTAIR exhibits a pro-oncogenic activity by modulating the cell cycle, and its main function of is to inhibit the expression of the target genes *via* H3K27 histone methylation and mediation of the PRC2 complex activity and to promote tumor progression (13, 24, 25). However, it is unclear whether HOTAIR is involved in the regulation of inflammation and immune escape in glioma cells. In the present study, we demonstrated the role of HOTAIR in the regulation of the NF-κB pathway, which is associated with inflammation and immunity. The analysis of protein spectrum data from HOTAIR knockdown cell lines revealed that HOTAIR expression was closely associated with the expression of NF-κB pathway proteins. *In vitro* experiments demonstrated that HOTAIR regulates the phosphorylation and nucleation of NF-κB, which was dependent on the epigenetic modulation of NF-κB negative regulators by HOTAIR. Furthermore, *in vivo* and *in vitro* experiments demonstrated that AQB inhibits tumor growth and reduces NF-κB and phospho-NF-κB levels in the xenograft model. Furthermore, AQB improved the immune sensitivity of tumor cells similarly to the effect exerted by a HOTAIR siRNA. These results provide evidence that HOTAIR regulates tumor cell inflammatory responses and immune escape *via* the NF-κB pathway in glioblastoma.

In a variety of malignancies with aberrant NF-κB activity, only a fraction of them are due to mutations in NF-κB genes. Abnormal activation of NF-κB in many malignant solid tumor cells may be due to inflammatory cytokine stimulation in the tumor microenvironment (18, 31). Herein, our data indicate that the lncRNA HOTAIR promotes NF-κB activity in malignant glioma cells, whereas its overexpression leads to aberrant NF-κB activation even without inflammatory stimulation. These findings suggest that the lncRNA HOTAIR is an essential “driver” of NF-κB signaling in malignant glioma cells. NF-κB is constitutively active in many tumor types and is considered a key factor in cancer development (18, 31). A number of negative regulators of the NF-κB pathway such as the deubiquitinases (DUB) A20 (32) and CYLD (33), ubiquitin ligase SOCS-1 (19), and a group of miRNAs (34), etc., have been identified as tumor suppressors. Many of these regulators are transcribed by NF-κB but participate in negative feedback loops to prevent a sustained or excessive activation of the NF-κB pathway (19). The activation and release of NF-κB depend on the phosphorylation of IκBα by the classical IKKα/IKKβ/CHUK complex. A cytoplasmic, NF-κB-interacting lncRNA reportedly blocks IκB phosphorylation and suppresses breast cancer metastasis (35). In our study, the lncRNA HOTAIR was implicated in the abnormal activation of the NF-κB pathway. Furthermore, protein spectrum data and *in vitro* experiments showed that HOTAIR activates the protein expression of the TNFα/NF-κB signaling complex and IκBα kinase complex. This abnormal activation may lead to high PD-L1 expression, which may block the tumor cell killing by T cells.

## Conclusions

In summary, our data demonstrated that HOTAIR activates the implicated in inflammatory responses NF-κB pathway in mesenchymal glioma cells and promotes immune escape *via* an aberrant PD-L1 expression. Furthermore, *in vivo* and *vitro* data showed that HOTAIR inhibits the NF-κB repressors UBXN1 *via* an H3K27me3 modification, which promotes IKKα/IKKβ/IKKγ-IκBα-induced NF-κB activation and transmission and forms an HOTAIR-NF-κB axis.

## Data Availability Statement

The data presented in the study are deposited in the iProX repository, accession number IPX0003692000.

## Ethics Statement

The animal study was reviewed and approved by Tianjin Medical University General Hospital Experimental Animal Welfare Ethics Committee.

## Author Contributions

YW, KY, and XL designed experiments, analyzed data and wrote the paper. YW, KY, WJ, JZ, YL, and YT performed experiments and contributed to writing. HW and CK supervised the study. All authors contributed to the article and approved the submitted version.

## Funding

This work was supported by grants from National Nature Science Foundation of China (82073322) and Tianjin Key R&D Plan of Tianjin Science and Technology Plan Project (20YFZCSY00360).

## Conflict of Interest

The authors declare that the research was conducted in the absence of any commercial or financial relationships that could be construed as a potential conflict of interest.

## Publisher’s Note

All claims expressed in this article are solely those of the authors and do not necessarily represent those of their affiliated organizations, or those of the publisher, the editors and the reviewers. Any product that may be evaluated in this article, or claim that may be made by its manufacturer, is not guaranteed or endorsed by the publisher.

## Abbreviations

LncRNAs, long noncoding RNAs; HOTAIR, HOX antisense intergenic RNA; PRC2, polycomb repressive complex 2; EZH2, enhancer Of zeste 2 polycomb repressive complex 2; NF-κB, nuclear factor-κB; IκBα, inhibitor of NF-κB alpha; IKK, IκB kinases; NKRF, NFκB repressing factor; PRKCQ, protein kinase C theta; UBXN1, ubiquitin-associated and ubiquitin regulatory X domain-containing protein 1; PD-L1, Programmed Cell Death 1 Ligand 1; TCGA, the cancer genome atlas; CGGA, Chinese Glioma Genome Atlas; KEGG, Kyoto encyclopedia of genes and genomes; SD, standard deviation.
