# Supplementary material for: HOTAIR Up-Regulation Activates NF-κB to Induce Immunoescape in Gliomas
Source: Front Immunol. 2021 Nov 23;12:785463. doi: 10.3389/fimmu.2021.785463 (PMC8649724; doi:10.3389/fimmu.2021.785463)
Supplement: Supplementary file 1 [file DataSheet_1.docx]

**Supplementary Table 1. Primers for quantitative real-time PCR analysis**

| Name | | Coding | | Anticoding |
| --- | --- | --- | --- | --- |
| XIAP | TCACTTGAGGTTCTGGTTGCAG | | TTGCAAAGCTTCTCCTCTTGC | |
| BCL-2 | GGATCCAGGATAACGGAGGC | | GGGCCAAACTGAGCAGAGTC | |
| IL-8 | GAGTGATTGAGAGTGGACCACACT | | AGACAGAGCTCTCTTCCATCAGAAA | |
| IL-1Β | CCACAGACCTTCCAGGAGAATG | | GTGCAGTTCAGTGATCGTACAGG | |
| TNFΑ | CTCTTCTGCCTGCTGCACTTTG | | ATGGGCTACAGGCTTGTCACTC | |
| PD-L1 | AGGAGTACCTTGGCTTTGCC | | GCCTTGCTCAGCCACAATTC | |
| NKRF | TCATCTTTGTGTGAAGCCAGT | | AGTCTGGAAATCACATGGTAGAAGT | |
| PRKCQ | TCCATCCACCCATTCTCAGC | | ACCAGTCATGGCACGAGAAG | |
| UBXN1 | AAGAGTTGTCAGCAGCACGA | | CCCACACTGCCACCATACTT | |
| HOTAIR | ATAGGCAAATGTCAGAGGGTT | | TCTTAAATTGGGCTGGGTC | |

**Supplementary Table 2. Primers used in ChIP assay**

| Name | Coding | Anticoding |
| --- | --- | --- |
| UBXN1 promotor 1 | ACGTCTGGTCAAAGAACCTT | TCCTCTCTTTCTTCACGCTC |
| UBXN1 promotor 2 | GGCAGGGGAAATGATGGATA | TAACCTGCCCACTCCATTAC |
| HOTAIR | GGAAAGCAGCAAAATGGGGG | GTTGTGGCCTATGCCTCCTT |
| GAPDH | TACTAGCGGTTTTACGGGCG | TCGAACAGGAGGAGCAGAGAGCGA |
| CD274 | ACTGAAAGCTTCCGCCGATT | GAGGAACAACGCTCCCTACC |

**Supplementary Table 3. Probes for ChIRP analysis**

| **Name** | **Coding** |
| --- | --- |
| hotair-1 | TGTGGAAGCTTTCGGATCAA |
| hotair-2 | TTAGGGACCTGAGGGTCTAA |
| hotair-3 | AAATCCGTTCCATTCCACTG |
| hotair-4 | AATAAAGACGCCCCTCCTTC |
| hotair-5 | TTTCAGCCTTTTCTCTGCCA |
| hotair-6 | GGTGTAATTGCTGGTTTAGG |
| hotair-7 | TAAACCTCTGTCTGTGAGTG |
| hotair-8 | AGGTTTTTCCAGCGTTCTCT |
| hotair-9 | ATTAATTAGCGCCTCCCAGT |
| hotair-10 | CTGTTTGGGCCTCCTAAAAT |
